# Key information providers, channels, and characteristics of Japanese consumers’ informed choices of over-the-counter medications

**DOI:** 10.1186/s40064-015-1549-7

**Published:** 2015-11-26

**Authors:** Makiko Hayashi, Sachiko Masuda, Hiromichi Kimura

**Affiliations:** Pharmaco-Business Innovation Laboratory, Graduate School of Pharmaceutical Sciences, The University of Tokyo, 7-3-1, Hongo, Bunkyo-ku, Tokyo, 113-0033 Japan

**Keywords:** Information, Information channel, Information characteristic, Information needs, Information provider, Informed choice, Over-the-counter medication, Self-medication

## Abstract

People need reliable information regarding over-the-counter medications (OTCs), so that they can independently make appropriate informed choices. The study aimed to identify the information providers and channels that have an impact on the purchase of OTCs, and to demonstrate the information needs of OTC purchasers, using these providers and channels, from the viewpoint of information characteristics such as specialty, objectivity, concreteness, comprehensiveness, individuality, and availability, focusing on the efficacy of OTCs and related safety information. A questionnaire survey of randomly sampled adults aged ≥20 was conducted at the Japan Drugstore Show 2012, hosted by the Japan Association of Chain Drug Stores. In this questionnaire, information was particularly limited to the efficacy and safety of OTCs. Multivariate logistic regression analysis was performed on data from 1743 respondents (1625 purchasers and 118 non-purchasers of OTCs) who obtained information on OTCs in their daily lives, to demonstrate the associations between the use of information providers and channels (predictor variables) and the purchase of OTCs (outcome variable), as well as between information characteristics valued by purchasers (predictor variables) and their use of these information providers or channels (outcome variables). Both the use of pharmacists as information providers and consultation at pharmacies as an information channel were positively associated with the purchase of OTCs (odds ratio [OR], 3.74; 95 % confidence interval [CI], 2.46–5.68; *P* < 0.001 and OR, 4.55; 95 % CI 2.92–7.11, *P* < 0.001, respectively), whereas both the use of family or friends using OTCs as information providers and family or friends as information channels were negatively associated with the purchase of OTCs (OR, 0.60; 95 % CI 0.40–0.90; *P* = 0.014 and OR, 0.55; 95 % CI 0.36–0.82; *P* = 0.004, respectively). OTC purchasers who valued individuality of information were more likely to use pharmacists (OR 2.00; 95 % CI 1.61–2.48; *P* < 0.001) and consultation at pharmacies (OR 1.98; 95 % CI 1.61–2.43; *P* < .001). In conclusion, individualized information provided by pharmacists on the efficacy and safety of OTCs during consultation at pharmacies can play the most important role in the informed choices of OTC purchasers.

## Background

### Self-medication in Japan

During the 20th century, along with socioeconomic development, lifestyle related diseases became the major cause of death in Japan and other advanced countries (The Ministry of Health, Labour and Welfare [MHLW] 2015). Consequently, the promotion of public health became an important issue for Japan (MHLW 2002a). Against this backdrop, the MHLW initiated “Healthy Japan 21” in March 2001, and the “Health Promotion Act” in August 2002 (MHLW 2002a; Sakurai 2003). These initiatives promulgated that people should have a duty to promote their own health. Along with changes in disease structure such as increase of adult diseases by progression of aging, repercussion of informed consent and pursuit of quality of life (QOL), more Japanese have become conscious of their health and medical care (MHLW 2007). “Self-care” that people do for themselves to establish and maintain health, prevent and deal with illness (The World Health Organization [WHO] 1998), and furthermore, “self-medication” that people select and use medicines by themselves to treat self-recognized illnesses or symptoms began to spread through the Japanese people (MHLW 2007). Currently, self-medication is a widespread practice in Japan (Shaku et al. 2015). With a view of improving the efficient use of medical resources, the MHLW released a report on the expected role of OTCs in self-medication in November 2002 (MHLW 2002b), which stated that broader use of OTCs should be recommended in self-medication, and that their role should include not only the treatment of minor diseases, but also prevention of diseases, early treatment of diseases, prevention of disease exacerbation, and self-examination of health status. Table 1 shows the recent legislative/non-legislative changes related to OTCs in Japan. Among various self-medication options, the use of OTCs is the most prevalent (Aoyama et al. 2012). In Japan, OTCs are classified into four categories, according to their risk levels: OTCs requiring guidance (post-marketing safety evaluation is incomplete), first-class OTCs (especially high risk), second-class OTCs (relatively high risk), and third-class OTCs (others). Consumers can purchase all the OTC categories at OTC pharmacies and out-of-hospital dispensing pharmacies; first-, second, and third-class OTCs at online pharmacies; and third-class OTCs at convenience stores and supermarkets. Recent market trends for OTCs in Japan have proven sluggish. In 2013, the market had shown a slight, 1.6 % growth (614 billion yen; Fuji Keizai 2014) since 2012 (604.3 billion yen), although it had decreased by 2.2 % since 2008 (627.7 billion yen; Fuji Keizai 2009). In contrast, the market for switch-OTCs in Japan, which had been 137.5 billion yen in 2005, reached 150 billion yen in 2011, and has been on an expansionary trend since then (Fuji Keizai 2015). The Japanese Pharmaceutical Affairs Law was revised in 2013; a new category was added to the OTC risk categories, “OTCs requiring guidance,” into which new switch-OTCs are classified. This revision of the trading system of OTCs would accelerate the switching of prescription drugs to OTCs. The market for switch-OTCs in Japan was expected to reach 162 billion yen in 2014, and to continue growing beyond 2015 (Fuji Keizai 2015).

**Table 1:** Recent legislative/non-legislative changes related to OTCs in Japan

Year	Recent legislative/non-legislative changes related to OTCs in Japan
2002	An online chain OTC pharmacy initiated online sales of OTCs
2009	OTCs were classified into 3 categories, according to their risk levels: first-class OTCs (especially high risk), second-class OTCs (relatively high risk), and third-class OTCs (others) The online sale of first- and second-class OTCs was prohibited The registered distributor qualification system was enforced Two online chain OTC pharmacies filed a lawsuit against the government, arguing for the ban on online OTC sales to be lifted
2013	The Supreme Court ruled that the ban on online OTC sales was illegal and, thus, invalid
2014	A new risk category (OTCs requiring guidance [post-marketing safety evaluation is incomplete]) was added to the OTC classification The online sale of all OTCs, except those requiring guidance, was officially permitted

*OTC* over-the-counter medication

### Consumers’ attitudes toward information on OTCs

With regard to OTCs, consumers tended to require efficacy (Sasaki et al. 2008; Tokyo metropolitan government 2011) and safety information (MHLW 1997; Mutebi et al. 2013; Raynor et al. 2007; Wazaify et al. 2005), more than any other information. OTC manufacturers indicate the efficacy and safety information of OTC products on their packages; however, a large number of people did not pay attention to written information accompanying medicines (Raynor et al. 2007). An eye-movement study revealed that Japanese consumers paid less attention to risk information appearing on the packages of OTCs than did Americans (Kawase et al. 2012). Pharmacies were the most common place for individuals to obtain information on OTC products (Eichenberg et al. 2015). Japanese consumers tended to want to consult with pharmacists when they purchased OTCs at pharmacies (Aoyama et al. 2012; Sasaki et al. 2008). These findings indicated that consumers might obtain information on the efficacy and safety of OTCs from information sources other than the OTC packages or other written instructions, perhaps from pharmacists at pharmacies.

### Aims of the study

The study aimed to identify information providers and channels of the efficacy and safety information of OTCs, that ultimately had an impact on Japanese consumers’ purchase of OTCs. The study also intended to reveal the information needs of OTC purchasers presenting at every information provider and channel, from the viewpoint of the following information characteristics, instead of information content: specialty, objectivity, concreteness, comprehensiveness, individuality, and availability, and then discussed appropriate methods of information provision that could support consumers’ informed choices of OTCs.

## Results

### Study subjects

1872 subjects responded to the questionnaires; a response rate of 93.6 % was achieved within a day. Of these individuals, 1743 completed the questionnaires. All of them indicated in the questionnaire that they obtained information regarding OTCs in their daily lives, and their data formed the basis of the analyses in this paper; 1625 (93.2 %) purchased OTCs in their daily lives (purchasers), and 118 (6.8 %) did not (non-purchasers).

### Information providers and the purchase of OTCs

Table 2 shows the associations between the use of information providers and the purchase of OTCs. The use of pharmacists as information providers was positively associated with the purchase of OTCs (odds ratios [ORs] 3.74; 95 % confidence interval [95 % CI] 2.46–5.68; *P* < 0.001), while the use of family or friends using OTCs was negatively associated with OTC purchase (OR 0.60; 95 % CI 0.40–0.90; *P* = 0.014). The utilization rate of OTCs among non-purchasers who used family or friends using OTCs as information providers was 71 % (data not shown).

**Table 2:** Associations between the use of information providers and the purchase of OTCs

Information provider (used: yes/no)	Multivariate logistic regression analysis^a^	
	OR^b^ (95 % CI)	*P*-value^c^
Pharmacists		
Yes	3.74 (2.46, 5.68)	<0.001
No	1.00 [Reference]	
OTC manufacturers		
Yes	1.13 (0.76, 1.67)	0.549
No	1.00 [Reference]	
Family or friends using OTCs		
Yes	0.60 (0.40, 0.90)	0.014
No	1.00 [Reference]	
Medical doctors		
Yes	0.77 (0.51, 1.17)	0.220
No	1.00 [Reference]	
Ordinary users of OTCs		
Yes	1.42 (0.87, 2.32)	0.157
No	1.00 [Reference]	
Public organizations		
Yes	1.07 (0.62, 1.83)	0.806
No	1.00 [Reference]	
Nurses		
Yes	1.09 (0.60, 1.98)	0.769
No	1.00 [Reference]	
Salespersons		
Yes	0.87 (0.45, 1.68)	0.682
No	1.00 [Reference]	
Mass communications		
Yes	1.80 (0.88, 3.69)	0.107
No	1.00 [Reference]	
Registered distributors		
Yes	1.59 (0.66, 3.82)	0.298
No	1.00 [Reference]	
Public health nurses		
Yes	0.51 (0.25, 1.02)	0.058
No	1.00 [Reference]	
OTC experts		
Yes	1.14 (0.42, 3.08)	0.803
No	1.00 [Reference]	
People of learning and experience		
Yes	0.66 (0.26, 1.69)	0.383
No	1.00 [Reference]	

The outcome and predictor variables were the purchase of OTCs (Yes/No) and the use of each information provider (Yes/No), respectively

*CI* confidence interval, *OR* odds ratio, *OTC* over-the-counter medication

^a^n = 1743

^b^Variables were mutually adjusted

^c^Statistically significant (*P* < 0.05)

### Valuable information characteristics and information providers

Among OTC purchasers, the proportions of those who considered concreteness, individuality, objectivity, availability, specialty, and comprehensiveness to be valuable for information on OTCs were 65.8, 45.8, 39.9, 32.7, 32.2, and 13.2 %, respectively (data not shown). Among the 13 information providers investigated, the five information providers used most often by OTC purchasers were pharmacists, OTC manufacturers, family or friends using OTCs, medical doctors, and ordinary users (utilization rate: 65.2, 60.8, 53.5, 44.7, and 26.6 %, respectively; data not shown). Table 3 shows the associations between information characteristics valued and the use of these five information providers in OTC purchasers. Those who considered specialty to be valuable for information on OTCs were more likely to use OTC manufacturers, medical doctors, or ordinary users of OTCs (OR, 1.50; 95 % CI 1.20–1.88; *P* < 0.001, OR, 2.12; 95 % CI 1.71–2.64; *P* < 0.001, or OR 1.29; 95 % CI 1.01–1.63; *P* = 0.039, respectively) for their information providers. Those considering objectivity valuable were more likely to use ordinary users of OTCs (OR, 1.57; 95 % CI, 1.25–1.97; *P* < 0.001); those who considered concreteness valuable were more likely to use family or friends using OTCs, medical doctors, or ordinary users of OTCs (OR 1.82; 95 % CI 1.47–2.25; *P* < 0.001, OR 1.35; 95 % CI 1.09–1.67; *P* = 0.006, or OR 1.76; 95 % CI 1.37–2.26; *P* < 0.001, respectively); those who considered comprehensiveness valuable were more likely to use OTC manufacturers or medical doctors (OR 1.53; 95 % CI 1.12–2.10; *P* = 0.008 or OR 1.41; 95 % CI 1.05–1.90; *P* = 0.024, respectively); those who considered individuality valuable were more likely to use pharmacists or medical doctors (OR 2.00; 95 % CI 1.61–2.48; *P* < 0.001 or OR 1.41; 95 % CI 1.15–1.73; *P* = 0.001, respectively) and unlikely to use OTC manufacturers (OR 0.71; 95 % CI 0.58–0.88; *P* = 0.001); and those who considered availability valuable were more likely to use OTC manufacturers, family or friends using OTCs, or ordinary users of OTCs (OR 1.27; 95 % CI 1.02–1.57; *P* = 0.033, OR 1.46; 95 % CI 1.18–1.80; *P* = 0.001, or OR 1.73; 95 % CI 1.37–2.18; *P* < 0.001, respectively).

**Table 3:** Associations between information characteristics valued and the use of information providers in OTC purchasers

Information characteristic (valued: yes/no)	Multivariate logistic regression analyses^a^									
	Pharmacists		OTC Manufacturers		Family or friends using OTCs		Medical doctors		Ordinary users of OTCs	
	OR^b^ (95 % CI)	*P*-value^c^	OR^b^ (95 % CI)	*P*-value^c^	OR^b^ (95 % CI)	*P*-value^c^	OR^b^ (95 % CI)	*P*-value^c^	OR^b^ (95 % CI)	*P*-value^c^
Specialty										
Yes	0.97 (0.78, 1.22)	0.818	1.50 (1.20, 1.88)	<0.001	0.85 (0.68, 1.05)	0.129	2.12 (1.71, 2.64)	<0.001	1.29 (1.01, 1.63)	0.039
No	1.00 [Reference]		1.00 [Reference]		1.00 [Reference]		1.00 [Reference]		1.00 [Reference]	
Objectivity										
Yes	1.14 (0.92, 1.42)	0.218	1.03 (0.83, 1.27)	0.793	1.21 (0.98, 1.48)	0.074	1.21 (0.98, 1.48)	0.074	1.57 (1.25, 1.97)	<0.001
No	1.00 [Reference]		1.00 [Reference]		1.00 [Reference]		1.00 [Reference]		1.00 [Reference]	
Concreteness										
Yes	0.88 (0.70, 1.10)	0.252	1.16 (0.94, 1.44)	0.172	1.82 (1.47, 2.25)	<0.001	1.35 (1.09, 1.67)	0.006	1.76 (1.37, 2.26)	<0.001
No	1.00 [Reference]		1.00 [Reference]		1.00 [Reference]		1.00 [Reference]		1.00 [Reference]	
Comprehensiveness										
Yes	1.17 (0.86, 1.60)	0.324	1.53 (1.12, 2.10)	0.008	1.08 (0.80, 1.45)	0.613	1.41 (1.05, 1.90)	0.024	1.35 (0.98, 1.87)	0.066
No	1.00 [Reference]		1.00 [Reference]		1.00 [Reference]		1.00 [Reference]		1.00 [Reference]	
Individuality										
Yes	2.00 (1.61, 2.48)	<0.001	0.71 (0.58, 0.88)	0.001	0.83 (0.68, 1.02)	0.073	1.41 (1.15, 1.73)	0.001	0.90 (0.72, 1.14)	0.383
No	1.00 [Reference]		1.00 [Reference]		1.00 [Reference]		1.00 [Reference]		1.00 [Reference]	
Availability										
Yes	1.20 (0.96, 1.50)	0.108	1.27 (1.02, 1.57)	0.033	1.46 (1.18, 1.80)	0.001	1.15 (0.93, 1.43)	0.192	1.73 (1.37, 2.18)	<0.001
No	1.00 [Reference]		1.00 [Reference]		1.00 [Reference]		1.00 [Reference]		1.00 [Reference]	

The outcome and predictor variables were the use of each information provider (Yes/No) and the valuing of each information characteristic (Yes/No), respectively

*CI* confidence interval, *OR* odds ratio, *OTC* over-the-counter medication

^a^n = 1625

^b^Variables were mutually adjusted

^c^Statistically significant (*P* < .05)

### Information channels and the purchase of OTCs

Table 4 shows the associations between the use of information channels and the purchase of OTCs. The use of consultation at pharmacies as an information channel was positively associated with the purchase of OTCs (OR 4.55; 95 % CI 2.92–7.11; *P* < 0.001), while the use of family or friends was negatively associated with the purchase of OTCs (OR 0.55; 95 % CI 0.36–0.82; *P* = 0.004). The utilization rate of OTCs among non-purchasers who used family or friends as information channels was 70 % (data not shown).

**Table 4:** Associations between the use of information channels and the purchase of OTCs

Information channel (used: yes/no)	Multivariate logistic regression analysis^a^	
	OR^b^ (95 % CI)	*P*-value^c^
Consultation at pharmacies		
Yes	4.55 (2.92, 7.11)	<0.001
No	1.00 [Reference]	
Family or friends		
Yes	0.55 (0.36, 0.82)	0.004
No	1.00 [Reference]	
Internet		
Yes	1.29 (0.86, 1.93)	0.213
No	1.00 [Reference]	
Consultation at medical institutions		
Yes	0.94 (0.63, 1.39)	0.750
No	1.00 [Reference]	
TV or radio		
Yes	1.05 (0.70, 1.59)	0.809
No	1.00 [Reference]	
Newspapers or advertising inserts		
Yes	0.74 (0.47, 1.16)	0.189
No	1.00 [Reference]	
Packages or package leaflets		
Yes	1.02 (0.63, 1.64)	0.943
No	1.00 [Reference]	
Books or magazines		
Yes	1.48 (0.72, 3.04)	0.293
No	1.00 [Reference]	
Customer services of OTC manufacturers		
Yes	0.93 (0.48, 1.80)	0.838
No	1.00 [Reference]	
Consultation services of public organizations		
Yes	1.14 (0.53, 2.46)	0.734
No	1.00 [Reference]	

The outcome and predictor variables were the purchase of OTCs (Yes/No) and the use of each information channel (Yes/No), respectively

*CI* confidence interval, *OR* odds ratio, *OTC* over-the-counter medication

^a^n = 1743

^b^Variables were mutually adjusted

^c^Statistically significant (*P* < 0.05)

### Valuable information characteristics and information channels

Among the 10 information channels investigated, OTC purchasers used consultation at pharmacies, family or friends, the Internet, consultation at medical institutions, and TV or radio as the five most used information channels (utilization rate: 55.9, 53.4, 42.8, 42.4, and 42.0 %, respectively; data not shown). Table 5 shows the associations between information characteristics valued and the use of these five information channels in OTC purchasers. Those who considered specialty valuable for information on OTCs were more likely to use the Internet or consultation at medical institutions (OR 1.95; 95 % CI 1.57–2.42; *P* < 0.001 or OR 1.75; 95 % CI 1.41–2.17; *P* < 0.001, respectively), and unlikely to use consultation at pharmacies (OR 0.79; 95 % CI 0.64–0.98; *P* = 0.030) for their information channels. Those who considered objectivity valuable were more likely to use family or friends, the Internet, or consultation at medical institutions (OR 1.23; 95 % CI 1.00–1.51; *P* = 0.047, OR 1.38; 95 % CI 1.12–1.70; *P* = 0.002, or OR 1.29; 95 % CI 1.05–1.59; *P* = 0.014, respectively). Those who considered concreteness valuable were more likely to use family or friends, the Internet, or television (TV) or radio (OR 1.81; 95 % CI 1.46–2.23; *P* < 0.001, OR 1.59; 95 % CI 1.28–1.97; *P* < 0.001, or OR 1.26; 95 % CI 1.02–1.56; *P* = 0.036, respectively), whereas those who considered individuality valuable were more likely to use consultation at pharmacies or at medical institutions (OR 1.98; 95 % CI 1.61–2.43; *P* < 0.001 or OR 1.51; 95 % CI 1.23–1.85; *P* < 0.001, respectively), and unlikely to use TV or radio (OR 0.72; 95 % CI 0.59–0.88; *P* = 0.002). Those who considered availability valuable were more likely to use family or friends, the Internet, or TV or radio (OR 1.44; 95 % CI 1.16–1.78; *P* = 0.001, OR 1.56; 95 % CI 1.28–1.97; *P* < 0.001, or OR 1.30; 95 % CI 1.06–1.61; *P* = 0.013, respectively).

**Table 5:** Associations between information characteristics valued and the use of information channels in OTC purchasers

Information characteristic (valued: Yes/No)	Multivariate logistic regression analyses^a^									
	Consultation at pharmacies		Family or friends		Internet		Consultation at medical institutions		TV or radio	
	OR^b^ (95 % CI)	*P*-value^c^	OR^b^ (95 % CI)	*P*-value^c^	OR^b^ (95 % CI)	*P*-value^c^	OR^b^ (95 % CI)	*P*-value^c^	OR^b^ (95 % CI)	*P*-value^c^
Specialty										
Yes	0.79 (0.64, 0.98)	0.030	0.84 (0.68, 1.04)	0.107	1.95 (1.57, 2.42)	<0.001	1.75 (1.41, 2.17)	<0.001	1.21 (0.98, 1.50)	0.080
No	1.00 [Reference]		1.00 [Reference]		1.00 [Reference]		1.00 [Reference]		1.00 [Reference]	
Objectivity										
Yes	1.14 (0.93, 1.40)	0.221	1.23 (1.00, 1.51)	0.047	1.38 (1.12, 1.70)	0.002	1.29 (1.05, 1.59)	0.014	0.88 (0.72, 1.08)	0.212
No	1.00 [Reference]		1.00 [Reference]		1.00 [Reference]		1.00 [Reference]		1.00 [Reference]	
Concreteness										
Yes	0.99 (0.80, 1.22)	0.895	1.81 (1.46, 2.23)	<0.001	1.59 (1.28, 1.97)	<0.001	1.18 (0.95, 1.46)	0.135	1.26 (1.02, 1.56)	0.036
No	1.00 [Reference]		1.00 [Reference]		1.00 [Reference]		1.00 [Reference]		1.00 [Reference]	
Comprehensiveness										
Yes	1.06 (0.79, 1.42)	0.711	1.00 (0.74, 1.34)	0.988	1.27 (0.94, 1.71)	0.120	1.21 (0.90, 1.63)	0.209	1.17 (0.88, 1.57)	0.284
No	1.00 [Reference]		1.00 [Reference]		1.00 [Reference]		1.00 [Reference]		1.00 [Reference]	
Individuality										
Yes	1.98 (1.61, 2.43)	<0.001	0.86 (0.70, 1.05)	0.146	0.93 (0.76, 1.15)	0.512	1.51 (1.23, 1.85)	<0.001	0.72 (0.59, 0.88)	0.002
No	1.00 [Reference]		1.00 [Reference]		1.00 [Reference]		1.00 [Reference]		1.00 [Reference]	
Availability										
Yes	1.01 (0.82, 1.25)	0.899	1.44 (1.16, 1.78)	0.001	1.59 (1.28, 1.97)	<0.001	0.99 (0.80, 1.23)	0.950	1.30 (1.06, 1.61)	0.013
No	1.00 [Reference]		1.00 [Reference]		1.00 [Reference]		1.00 [Reference]		1.00 [Reference]	

The outcome and predictor variables were the use of each information channel (Yes/No) and the valuing of each information characteristic (Yes/No), respectively

*CI* confidence interval, *OR* odds ratio, *OTC* over-the-counter medication

^a^n = 1625

^b^Variables were mutually adjusted

^c^Statistically significant (*P* < 0.05)

## Discussion

### Novelty of the study

As far as we know, the current study is the first investigation to reveal the information needs of Japanese consumers regarding the efficacy and safety of OTCs by focusing on the features of the information rather than the information content. As mentioned above, with regard to OTCs, several previous studies have investigated the information sources that they used throughout their purchasing behavior. The current study focused on the step at which consumers chose and purchased specific OTCs based on certain information, seeking to address the following questions: (1) Did pharmacists as information providers and consultation at pharmacies as an information channel ultimately influence consumers’ choices regarding the purchase of specific OTCs? (2) What about other information providers and channels? (3) What information characteristics did consumers expect from their information providers and channels with regard to OTCs? The study identified information providers and channels that ultimately had an impact on the purchase of OTCs by Japanese consumers; one of the distinctive features of this study was that it investigated information sources by dividing them into information providers and channels. Information providers, each bringing different levels of credibility and expertise, should provide consumers with sufficient information to meet their information needs in order to give significant support to their informed choices regarding OTCs. The study also revealed the information needs of OTC purchasers at every information provider and channel they used, from the viewpoint of the features of the information.

### Key information providers, channels and characteristics

Using descriptive statistics, previous studies in Ireland (Wazaify et al. 2005) and in Japan (Aoyama et al. 2012) demonstrated that recommendations by pharmacists most frequently influenced consumers’ choice of OTCs. The current study obtained similar results, using inferential statistics. It demonstrated that information provided by pharmacists had a positive impact on Japanese consumers’ purchase of OTCs. As for information channels, a nationwide representative survey in Germany (Eichenberg et al. 2015) reported pharmacies to be the most common place from which consumers obtained information about OTCs. This survey, however, indicated neither the information channel (e.g., consultation and product packages) utilized by consumers at pharmacies nor whether or not the information obtained at pharmacies had an actual impact on consumers’ choices regarding the purchase of specific OTCs. The current study revealed that consultation at pharmacies was the most commonly used information channel for OTC purchasers, and that the information provided through this channel had a positive impact on consumers’ purchase of OTCs. A published systematic review (Raynor et al. 2007) reported interesting results. Most patients did not value the written information on medicines, which manufacturers had prepared according to strict regulations; however, they valued the information if it was tailored to their individual circumstances and illness. The findings indicated that patients typically required individualized, rather than statutory information, when they used such information for decision-making. The current study obtained similar results; OTC purchasers who used pharmacists as information providers and who used consultation at pharmacies as an information channel were more likely to value individualized information than were purchasers who did not use them. These findings indicate that it may be preferable for pharmacists to provide OTC purchasers mainly with information tailored to their individual conditions, constitution, lifestyles, and so on, during their consultation at pharmacies. The study also revealed that OTC purchasers who used consultation at pharmacies were less likely to require specialized information, suggesting that during consultation at pharmacies, purchasers should be provided mainly with individualized information, rather than specialized information containing scientific knowledge, such as action mechanisms of active ingredients of OTCs. OTC purchasers who needed specialized information were more likely to use other information channels, such as the Internet and medical institutions.

### Family or friends using OTCs as information providers and family or friends as information channels

This study also revealed that the information provided by family or friends using OTCs and the information provided through family or friends had a negative impact on consumers’ purchase of OTCs. The vast majority of non-purchasers who used such information, however, utilized OTCs, indicating that perhaps they entrusted the purchase of OTCs that they needed to someone else, rather than that some negative information provided by family or friends using OTCs or through family or friends made them give up purchasing OTCs. This “entrustment of purchasing OTCs” may be the source of the negative impact on the purchase of OTCs mentioned above. Family or friends using OTCs made up one of the five information providers used most often by OTC purchasers, and those who valued the concreteness or availability of information tended to use those information providers, rather than pharmacists. We assume that OTC purchasers might use family or friends using OTCs as information providers, when they want to know the efficacy and safety of OTCs actually and personally experienced by the latter, instead of general explanations. They may also use these individuals when they want to know concrete features of OTCs, such as the shape, size, taste, and smell thereof, and the ease with which OTCs can be swallowed, along with the efficacy and safety of OTCs; when they want to know the efficacy and safety of household OTCs; or when they quickly want some reference information, before getting individualized information from pharmacists at pharmacies.

### Registered distributors as information providers

Like pharmacists, registered distributors, who are qualified to sell second- and third-class OTCs, have opportunities to come face-to-face with OTC purchasers, and to have two-way communication with them at pharmacies; however, information provided by them didn’t have significant impact on their purchase of OTCs. The registered distributor system was enforced in 2009, so, perhaps that system was not well-recognized by OTC purchasers at the time of the survey; a 2011 survey in Tokyo, Japan (Tokyo metropolitan government 2011), reported that more than half of the OTC purchasers investigated did not know the registered distributor system.

### Medical doctors as information providers

Medical doctors can provide people with face-to-face two-way communication mainly at medical institutions, and just like pharmacists, they tended to be used by OTC purchasers who considered individuality a valuable information characteristic; however, information provided by them didn’t have significant impact on consumers’ purchase of OTCs. Although OTC purchasers who valued various information characteristics as well as individuality were more likely to use medical doctors, information provided by them, even individualized information, might not have had enough influence to lead people to purchase OTCs. A Patient Attitude Questionnaire in the West Midlands (Bradley et al. 1998) demonstrated that patients generally expressed positive attitudes regarding their doctors’ suggestions to try OTCs. It is probable that medical doctors might have an impact on consumers’ decisions to substitute prescription drugs with OTCs.

### Consultation at medical institutions as an information channel

Consultation at medical institutions can provide people with face-to-face two-way communication with medical personnel, and can provide them with individualized information; indeed, OTC purchasers who considered individuality to be a valuable information characteristic were more likely to use this information channel. However, information provided through consultation at medical institutions, even individualized information, didn’t have a significant impact on their purchase of OTCs. Combining these findings with those of the analyses focusing on pharmacists, medical doctors and consultation at pharmacies described above, it may be concluded that the purchase of OTCs is driven by “individualized information” provided by “face-to-face two-way communication” “at sites selling OTCs.”

### The Internet as an information channel

The results of the study demonstrated that information provided through the Internet didn’t have a significant impact on the purchase of OTCs. Those respondents who used the Internet for OTC purchases were more likely to require specialized, objective, concrete, or easily available information rather than individualized information at the time of the survey, indicating that the Internet served as a one-way communication tool to obtain general information. The Japanese Pharmaceutical Affairs Law was revised in June 2014, following which the online sale of most OTCs was officially permitted. The removal of the ban on online sales should enhance consumers’ convenience in purchasing OTCs. A previous survey in Germany (Holtgräfe and Zentes 2012) reported that the preferred use of the Internet as an OTC information source positively influenced the use of online pharmacies as purchase channels for OTCs. The current study showed that nearly half of OTC purchasers used the Internet as an information channel, and a questionnaire survey of Japanese consumers (Nakao et al. 2015) reported that 3.8 % of consumers used online pharmacies following the lifting of the ban on online sales. Taken together, these findings suggest that more consumers would use online pharmacies in the near future. When the survey was conducted, the utilization rate of the Internet as an information channel for OTCs was significantly lower than that of consultation at pharmacies. With the popularization of Internet OTC sales, purchasers’ use of online information will only increase, and the Internet will become a more important information channel in addition to functioning as a purchasing channel. Furthermore, it’s possible that the characteristics of Internet information sought by OTC purchasers might change; they might request online pharmacies to provide them with individualized information through two-way communication to make informed choices regarding OTCs.

### TV or radio as an information channel

TV or radio is a convenient information channel, and OTC purchasers who used them were more likely to value availability of information rather than individuality. TV or radio, however, did not have enough influence to lead consumers to purchase OTCs. We assume that information obtained through TV or radio, especially product commercials, would play a role in making consumers aware of OTC products, based on a Japanese questionnaire survey’s report (Aoyama et al. 2012) that, although OTC advertisements had a powerful effect on the perceptions of consumers of a low socioeconomic status, consumers did not rely solely on the information garnered from such advertisements, when choosing a product.

The findings of the current study also suggest that the information characteristics required by OTC purchasers differ according to both information providers and channels.

### Limitations of the study

The limitations of the study included the representativeness of the study subjects. OTC purchasers were in the majority. Most of the subjects could have been residents of a metropolitan area, and some subjects could have used OTC pharmacies (so-called “drugstores”) relatively more often because the study subjects were the visitors at the Japan Drugstore Show 2012, held in Chiba Prefecture. We, however, did not think that the study setting had a major influence on our findings, because Japanese consumers generally purchased OTCs at OTC pharmacies, as reported by several consumer surveys in Japan (MyVoice Co., Inc. 2012; Tokyo metropolitan government 2011; Tachi et al. 2015). An earlier survey study in Japan reported that more than half of high school students who used OTCs utilized them by their own decision (Anraku et al. 2011). As the current study included subjects aged ≥20, it is necessary to include younger people in the study sample to investigate their in situ use of information for OTCs. Another limitation was that the study didn’t consider consumers’ non-demographic/lifestyle factors that could influence their purchasing of OTCs. Associations between consumers’ use of OTCs and their non-demographic/lifestyle factors have been demonstrated in previous studies (Conn 1991; Morales-Suárez-Varela et al. 2009; Villako et al. 2012). Associations between consumers’ socioeconomic status and their decisions to use either prescription medications or OTCs have also been indicated (Aoyama et al. 2012; Nielsen et al. 2003; Sato et al. 2011). Although it is possible that some of these factors have a direct or indirect influence on consumers’ choices of information providers and channels, as well as on their information needs and their purchases of OTCs, no study investigating such possibilities has been reported to date. Further study should be conducted considering consumers’ socioeconomic status and non-demographic/lifestyle factors that may influence their OTC-related behaviour.

## Conclusion

This study demonstrated that pharmacists as information providers, consultation at pharmacies as an information channel, and the individualized information they provided played an important role in OTC purchasers’ informed choices about OTCs. Moreover, key information characteristics for OTC purchasers differed according to information providers and channels. The findings give information providers a useful clue to distribute more appropriate information that may facilitate consumers to make more informed choices regarding OTCs through suitable information channels.

## Methods

### Questionnaire

A self-developed questionnaire was prepared; it included respondents’ demographic information, such as gender and age, the purchase and utilization of OTCs, and the following questions: (1) Among the information providers below, which do you usually use to obtain information on OTCs? (2) Among the information channels below, which do you usually use to obtain information on OTCs? and (3) Among the information characteristics below, which do you consider valuable for information on OTCs? In this questionnaire, “information” was particularly limited to the efficacy and safety of OTCs. Thirteen information sources and 10 information channels were investigated. The information sources were OTC manufacturers, public organizations (e.g. MHLW, local health authorities), ordinary users of OTCs, family or friends using OTCs, mass communications, OTC experts, people of learning and experience (e.g. professors, academic researchers), medical doctors, pharmacists, registered distributors, nurses, public health nurses, and salespersons. The information channels were customer services of OTC manufacturers, consultation services of public organizations, consultation at pharmacies, the Internet, consultation at medical institutions, TV or radio, newspapers or advertising inserts, books or magazines, packages or package leaflets, and family or friends. For information characteristics, 6 properties were defined: “specialty” (specialized information about OTCs, including scientific knowledge such as action mechanisms of active ingredients), “objectivity” (fact-based scientific and objective information furnished by non-stakeholders), “concreteness” (concrete features of OTCs based on users’ real-life experience), “comprehensiveness” (information that covers a wide range of knowledge of every OTC product), “individuality” (information tailored to consumers’ individual conditions, constitution, lifestyles and so on), and “availability” (information easily obtained at any time and place).

### Execution of a questionnaire survey

A face-to-face, self-completed questionnaire survey was conducted at the Japan Drugstore Show 2012 on March 17, 2012 at Makuhari in Chiba Prefecture, Japan. The annual Japan Drugstore Show, hosted by the Japan Association of Chain Drug Stores, is one of the biggest exhibitions of healthcare in Asia with more than 120,000 visitors. 2000 questionnaires were randomly distributed to the visitors aged ≥20, who filled them out anonymously. The filled-out questionnaires were collected on the same day. Respondents who answered all the questions were identified as valid respondents. Among these, those who indicated in the questionnaires that they did not obtain information regarding OTCs in their daily lives were excluded from the analysis.

### Statistical analyses

Multivariate logistic regression analyses were performed to evaluate associations between the use of information providers or channels and the purchase of OTCs; the outcome and predictor variables were the purchase of OTCs (Yes/No) and the use of each information provider or channel (Yes/No), respectively. Next, the relationship between OTC purchasers’ valuing of OTC information characteristics and their use of each information provider or channel was evaluated using the same approach; the outcome and predictor variables were the use of each information provider or channel (Yes/No) and the valuing of each information characteristic (Yes/No), respectively. The results are presented as estimated ORs with respective 95 % CIs and *P*-values. All statistical evaluations were considered significant if *P* < 0.05. The platform for the statistical analyses was Ekuseru-Toukei 2012 (Social Survey Research Information Co., Ltd.).
